# Hobit expression by a subset of human liver-resident CD56^bright^ Natural Killer cells

**DOI:** 10.1038/s41598-017-06011-7

**Published:** 2017-07-27

**Authors:** Sebastian Lunemann, Gloria Martrus, Hanna Goebels, Tobias Kautz, Annika Langeneckert, Wilhelm Salzberger, Martina Koch, Madeleine J. Bunders, Björn Nashan, Klaas P. J. M. van Gisbergen, Marcus Altfeld

**Affiliations:** 1 Department of Virus Immunology, Heinrich Pette Institute, Leibniz Institute for Experimental Virology, Hamburg, Germany; 2 Department of Hepatobiliary Surgery and Transplantation, University Medical Center Hamburg, Hamburg, Germany; 3 Department of Experimental Medicine, Academic Medical Center, University of Amsterdam, Amsterdam, Netherlands

## Abstract

Immune responses show a high degree of tissue specificity shaped by factors influencing tissue egress and retention of immune cells. The transcription factor Hobit was recently shown to regulate tissue-residency in mice. Whether Hobit acts in a similar capacity in humans remains unknown. Our aim was to assess the expression and contribution of Hobit to tissue-residency of Natural Killer (NK) cells in the human liver. The human liver was enriched for CD56^bright^ NK cells showing increased expression levels of the transcription factor Hobit. Hobit^pos^ CD56^bright^ NK cells in the liver exhibited high levels of CD49a, CXCR6 and CD69. Hobit^pos^ CD56^bright^ NK cells in the liver furthermore expressed a unique set of transcription factors with higher frequencies and levels of T-bet and Blimp-1 when compared to Hobit^neg^ CD56^bright^ NK cells. Taken together, we show that the transcription factor Hobit identifies a subset of NK cells in human livers that express a distinct set of adhesion molecules and chemokine receptors consistent with tissue residency. These data suggest that Hobit is involved in regulating tissue-residency of human intrahepatic CD56^bright^ NK cells in a subset of NK cells in inflamed livers.

## Introduction

The quality of immune responses is influenced by a plethora of factors. There is mounting evidence that tissues are shaping immune responses to serve their specific needs through interactions between immune and tissue cells. Tissue homing, retention and egress of immune cells are important in ensuring that the correct immune microenvironment for each tissue is established and maintained. The underlying mechanisms influencing tissue specificity and residency are slowly being unraveled and will improve our understanding of this essential part of immunology.

Natural Killer (NK) cells are part of the innate immune system and play a pivotal role in the early control of infections^1^ and malignancies^2^. NK cells can be divided into two main subsets of CD56^dim^ and CD56^bright^ NK cells, based on their expression of CD56 and CD16^3^. In general, CD56^bright^ NK cells act by producing cytokines, while CD56^dim^ NK cells exert their effector functions through secretion of perforin and granzyme^2^. In the peripheral blood, CD56^dim^ NK cells make up roughly 90% of the NK cells pool, with CD56^bright^ NK cells contributing the remaining 10%. In contrast, CD56^bright^ NK cells represent the dominant population in lymphoid and non-lymphoid tissues^4^, and are also found in increased frequencies in inflamed and cancer tissues^5^. Tissue-resident NK cells have now been identified in uterus, liver and lymphoid tissues^4, 6^, and appear to play important roles not only in the defense against foreign pathogens and cancers^4^, but also in tissue remodeling and regeneration^4, 7^. While in mice several markers have been identified to define tissue-resident NK cells and it was shown that these tissue-resident NK cells are not circulating through the periphery^8^, the factors regulating tissue residency in humans are less well defined^9^.

Recent studies in humans have shown that some of the markers used to identify tissue-resident NK cells in murine livers, namely CD49a^9^ and CXCR6^10, 11^, are also expressed on human NK cells within the liver. Additionally, it was shown that CXCR6+ NK cells in human livers exhibited an Eomes^hi^ T-bet^lo^ phenotype^10, 11^, which is in contrast to the Eomes^lo^ T-bet^int^ phenotype initially described for CD49a+ NK cells in the liver^9^. This might suggest that several heterogeneous subsets of liver-resident NK cells exist in human livers. Further evidence for this was provided by a recent study describing CD49e as a marker almost exclusively expressed on NK cells derived from the human blood, whereas more than 50% of NK cells in the liver lack expression of CD49e^12^. A subset of Eomes^hi^ NK cells in human livers was shown to persist for up to 13 years in a transplantation setting^13^. However, if and how the turn-over of these liver-resident NK cell subsets is shaped by different transcriptional programs remains unknown. In mice, it was recently described that the transcription factor Hobit (homolog of Blimp1 in T cells or ZNF683), a zinc finger protein, acts in concert with Blimp-1 to serve as a master regulator of tissue-residency for lymphocytes^14^. Hobit was initially found to regulate NKT cell effector differentiation^15^ and subsequently also used to identify effector-type lymphocytes in humans^16^. Hobit acts together with Blimp-1 in regulating expression of genes involved in tissue retention and egress^14^, thereby shaping the lymphocyte compartment of the tissue, and Hobit knockout mice exhibited less tissue-resident NK cells in their liver^14^. Whether Hobit is also playing a role in regulating tissue-residency of human NK cells remains unknown. Here we investigated Hobit expression by human NK cells and its role in regulating tissue-residency of intrahepatic NK cells.

## Results

### CD56^bright^ NK cells are enriched in human liver tissue

**Figure 1:**
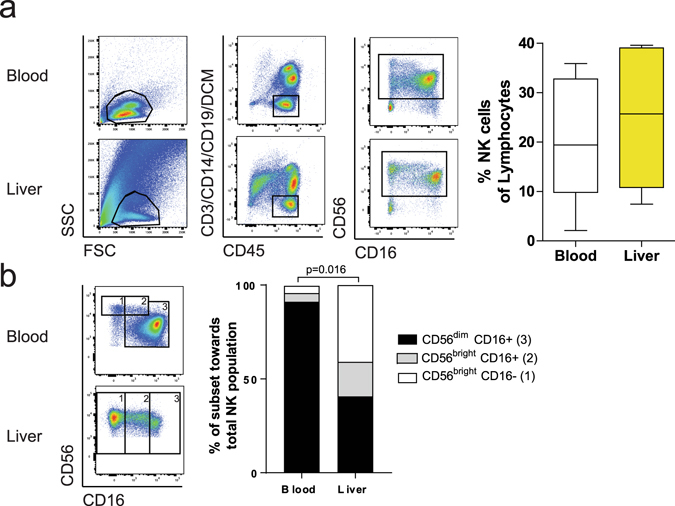
CD56^bright^ NK cells are enriched in human liver tissue. (**a**) Gating strategy (left panel) for identification of NK cells in peripheral blood (upper row) and liver tissue (lower row). Summarization of the data (right panel) comparing frequency of NK cells in blood and liver from matched patient samples (n = 7). Box and whiskers (Tukey) plots was used. (**b**) Identification of CD56^dim^ (3), CD56^bright^ CD16+ (2) and CD56^bright^ CD16- (1) NK cells (left panel) in peripheral blood (upper plot) and liver tissue (lower plot). Summarization of the data (right panel) comparing CD56^dim^,CD56^bright^ CD16+ and CD56^bright^ CD16- NK cells in the blood and liver of matched patient samples (n = 7). Bars show the median for all individuals, Wilcoxon matched pair sign rank test was used to determine statistical differences.

Matched liver and blood samples were obtained from individuals undergoing liver transplantation at the Department of Hepatobiliary and Transplant Surgery of the University Medical Center Hamburg. Basic clinical data of the study cohort is summarized in the supplement (Suppl. Table 1). All individuals had advanced liver disease requiring liver transplantation, including alcoholic liver disease, hepatitis C infection, hepatocellular carcinoma and cholangiocarcinoma. Flow cytometric analysis of the samples revealed that the overall frequency of bulk NK cells within the lymphocyte population was slightly higher in human liver samples (Fig. 1a) compared to matched peripheral blood. While this increase was not significant, the distribution of NK cell subsets was notably shifted between liver and blood samples (Fig. 1b), with a marked increase of both CD56^bright^ CD16- and CD56^bright^ CD16+ NK cells and a corresponding decrease of CD56^dim^ CD16+ NK cells (p = 0.016) in liver tissues.

### Increased frequency and expression of Hobit on CD56^bright^ liver NK cells

**Figure 2:**
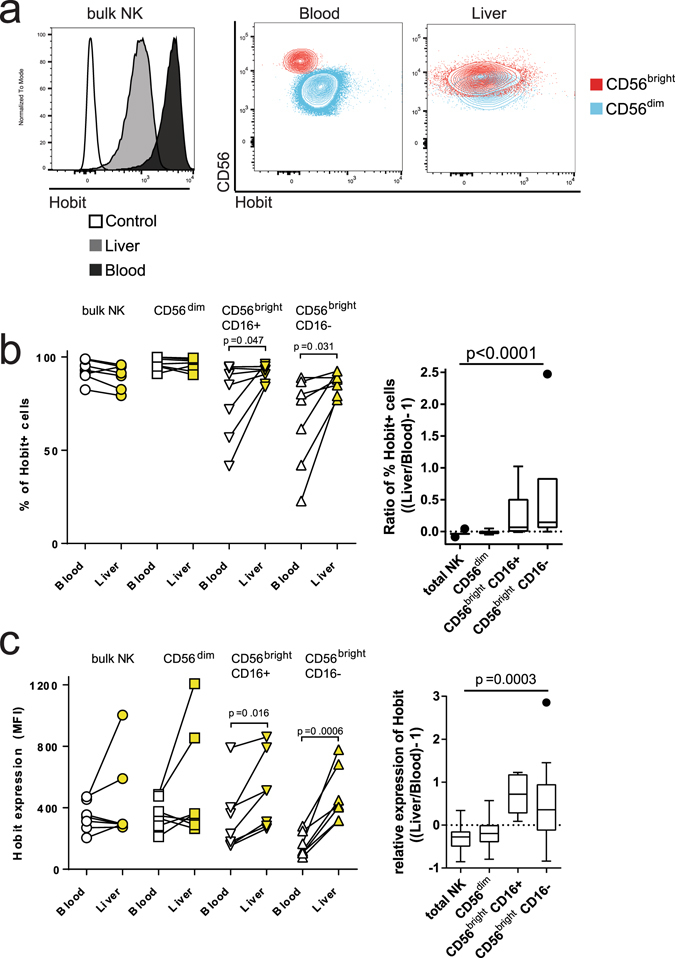
Increased frequencies and expression of Hobit on CD56^bright^ liver NK cells. (**a**) Histogram (left panel) showing Hobit expression on bulk NK cells in the blood (dark grey), the liver (light grey) and a FMO control (white). Contour plots showing the expression of Hobit on CD56^dim^ (blue) and CD56^bright^ (red) NK cells in the blood and liver. (**b**) Frequency (left panel) of Hobit^pos^ bulk, CD56^dim^,CD56^bright^ CD16+ and CD56^bright^ CD16- NK cells in the blood (open symbols) and liver (yellow symbols). Ratio of the frequency (right panel) of Hobit^pos^ cells in each subset between liver and peripheral blood. (**c**) Expression (left panel) of Hobit on bulk, CD56^dim^, CD56^bright^ CD16+ and CD56^bright^ CD16- NK cells in the blood (open symbols) and liver (yellow symbols). Ratio of the expression (right panel) of Hobit in each subset between liver and peripheral blood samples. Individual data points are blotted in the graphs on the left side. Box and whiskers (Tukey) was used to depict the expression ratio between liver and blood. Wilcoxon matched pair sign rank test was used to determine statistical differences in the scatter plots and Friedman test was used for box plots.

Next, we investigated the expression of the transcription factor Hobit in peripheral blood and liver-derived NK cells. Hobit was expressed on bulk liver-derived NK cells, albeit to a lower level than in matched blood samples (Fig. 2a). While almost all bulk and CD56^dim^ NK cells, independent of the compartment they were derived from, expressed Hobit (Fig. 2b), CD56^bright^ CD16^neg^ liver NK cells contained a significantly higher frequency of Hobit^pos^ cells when compared to CD56^bright^ CD16^neg^ NK cells derived from the blood (Fig. 2b, p = 0.031). Furthermore, these CD56^bright^ NK cells in the liver also expressed significantly higher levels of Hobit compared to their blood counterparts (Fig. 2c, p = 0.0006). CD56^bright^ CD16^pos^ NK cells in the liver showed an intermediate phenotype, both for the %-Hobit+ (Fig. 2b) and expression levels of Hobit (Fig. 2c), when compared to CD56dim and CD56bright CD16- NK cells. Taken together, these data demonstrate that CD56^bright^ NK cells represent a large fraction of the NK cells in the liver, and that these CD56^bright^ NK cells contain a higher frequency of Hobit^pos^ cells expressing higher levels of Hobit compared to CD56^bright^ NK cells in the peripheral blood.

### Hobit^pos^ CD56^bright^ liver NK cells express markers related to tissue residency

**Figure 3:**
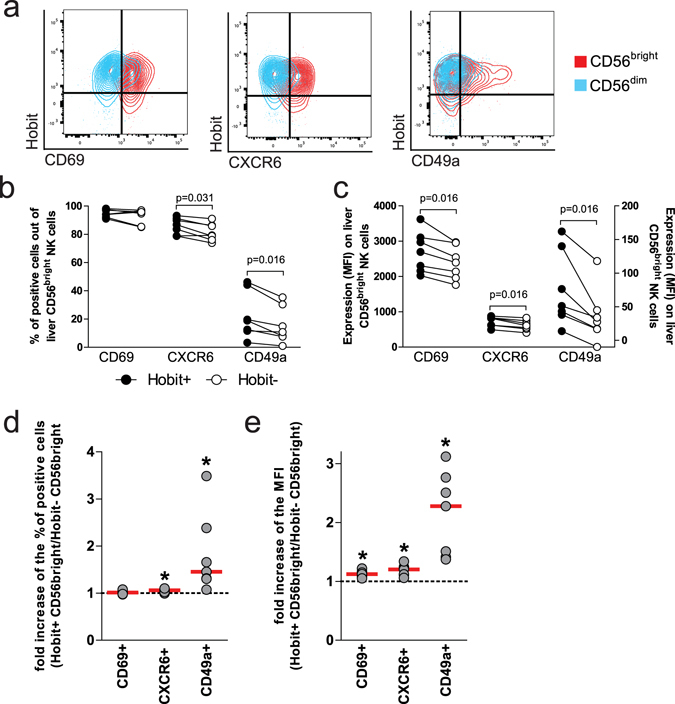
Hobit^pos^ CD56^bright^ liver NK cells express high levels of markers related to tissue residency. (**a**) Contour plots showing expression CD69 (left plot), CXCR6 (middle plot) and CD49a (right plot) compared to Hobit on CD56^bright^ (red) and CD56^dim^ (blue) NK cells in the liver. Frequency (**b**) and expression levels (**c**) of CD69, CXCR6 and CD49a comparing Hobit^pos^ (full symbols) to Hobit^neg^ (open symbols) CD56^bright^ NK cells from the liver. (**d**) Fold change (left panel) of CD69-, CXCR6- and CD49a-positive CD56^bright^ liver NK cells comparing Hobit^pos^ to Hobit^neg^ cells. (**e**) Fold increase of the MFI (right panel) of CD69, CXCR6 and CD49a on CD56^bright^ liver NK cells comparing Hobit^pos^ with Hobit^neg^ cells. Wilcoxon matched pair sign rank test was used to determine statistical differences in the scatter plots.

To determine whether Hobit expression was associated with the expression of other markers previously linked to tissue-residency of NK cells, we subsequently investigated the expression of the surface markers CD69, CXCR6, and CD49a on Hobit^pos^ NK cells^9–11^. We observed that CD69, CXCR6 and CD49a were all expressed on liver-derived NK cells, with higher levels on CD56^bright^ NK cells compared to CD56^dim^ NK cells (Fig. 3a). Furthermore, liver Hobit^pos^ CD56^bright^ NK cells contained a significantly higher frequency of CXCR6+ (p = 0.031) and CD49a+ (p = 0.016) cells (Fig. 3b and d), and exhibited a higher expression of CD69, CXCR6 and CD49a (p = 0.016 for all comparisons) compared to Hobit^neg^ CD56^bright^ NK cells (Fig. 3c and e). Overall, Hobit^pos^ CD56^bright^ NK cells in the liver showed a significantly higher expression of adhesion molecules and chemokine receptors related to tissue-residency compared to Hobit^neg^ CD56^bright^ NK cells.

### Altered expression of transcription factors in Hobit^pos^ CD56^bright^ liver NK cells

**Figure 4:**
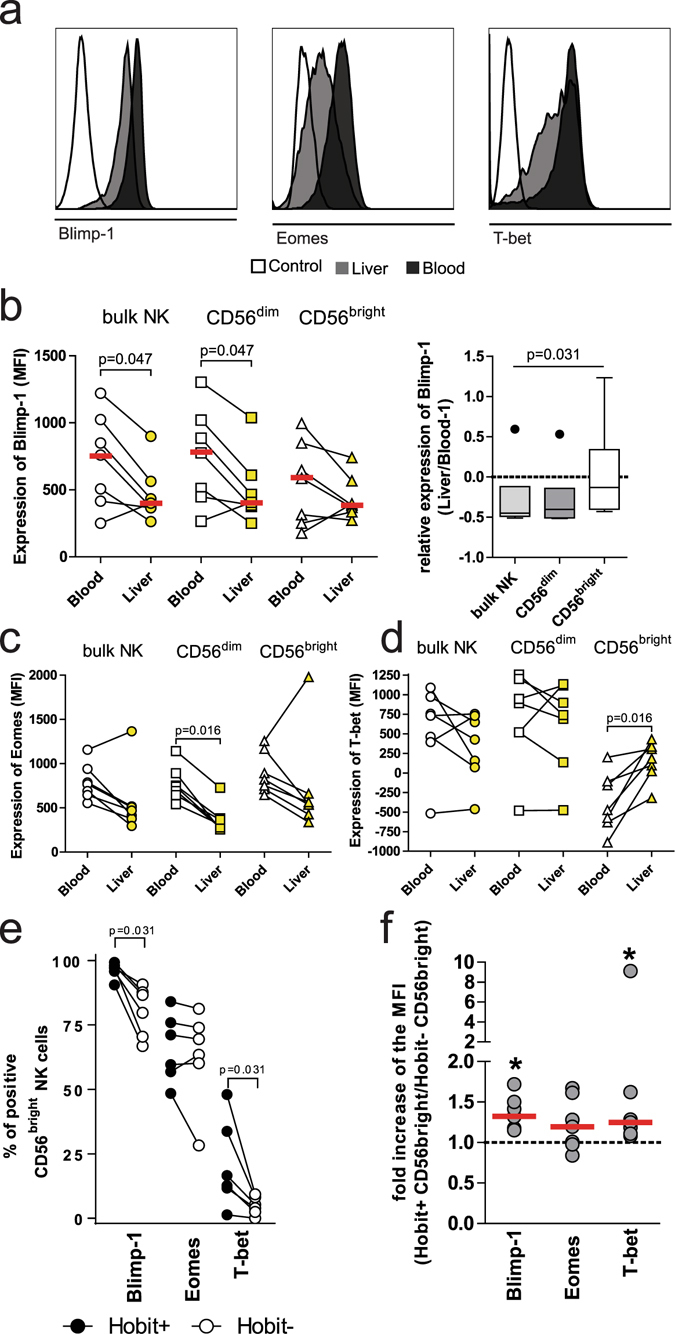
Altered expression of transcriptions factors in Hobit^pos^ CD56^bright^ liver NK cells. (**a**) Histogram showing expression of Blimp-1 (left panel), Eomes (middle panel) and T-bet (right panel) on bulk NK cells in the blood (dark grey) compared to the liver (light grey) and a FMO control (white). (**b**) Expression (left panel) of Blimp-1 on bulk, CD56^dim^ and CD56^bright^ NK cells in the peripheral blood (open symbols) and liver (yellow symbols). Ratio of the expression (right panel) of Blimp-1 in each subset between Liver and Blood. (**c**) Expression of Eomes on bulk, CD56^dim^ and CD56^bright^ NK cells in the blood (open symbols) and liver (yellow symbols). (**d**) Expression of T-bet on bulk, CD56^dim^ and CD56^bright^ NK cells in the blood (open symbols) and liver (yellow symbols). (**e**) Frequency of Blimp-1^pos^, Eomes^pos^ and T-bet^pos^ cells among CD56^bright^ liver NK cells comparing Hobit^pos^ (black symbol) with Hobit^neg^ (open symbol). (**f**) Fold change of the expression (MFI) of Blimp-1, Eomes and T-bet on CD56^bright^ liver NK cells comparing Hobit^pos^ with Hobit^neg^ cells. Wilcoxon matched pair sign rank test was used to determine statistical differences in the scatter plots and Friedman test was used for box plots.

Following the observation that Hobit^pos^ NK cells in the liver expressed markers of tissue-residency, we next assessed whether other transcription factors were also differentially expressed on CD56^bright^ NK cells in human livers. We therefore analyzed the expression of Blimp-1, T-bet and Eomes comparing bulk, CD56^dim^ and CD56^bright^ NK cells (Fig. 4a) derived from either peripheral blood or matched liver tissue. Blimp-1 was decreased in liver-derived bulk and CD56^dim^ NK cells compared to peripheral blood (Fig. 4b, left panel, p = 0.047, respectively). While the expression of Blimp-1 showed a tendency to be lower in liver-derived CD56^bright^ NK cells compared to peripheral blood (Fig. 4b, left panel, p = 0.3), this decrease in Blimp-1 expression was less pronounced compared to the relative decrease for bulk and CD56^dim^ NK cells, as reflected by the ratios of Blimp-1 expression between liver- and blood-derived NK cells (Fig. 4b, right panel, p = 0.03﻿1). Eomes expression was lower on liver-derived bulk, CD56^dim^ and CD56^bright^ NK cells, albeit only reaching significance for the CD56^dim^ NK cells from the liver (Fig. 4c, p = 0.016). T-bet was expressed at similar levels among bulk and CD56^dim^ NK cells comparing to blood and liver NK cells, but was significantly increased in liver-derived CD56^bright^ NK cells (Fig. 4d, p = 0.016). We subsequently focused the analysis of the expression of these different transcription factors on the Hobit^pos^ CD56^bright^ NK cell population in the liver, and observed higher frequencies of Blimp-1^pos^ and T-bet^pos^ cells compared to to Hobit^neg^ CD56^bright^ NK cells (Fig. 4e, p = 0.031, respectively). Expression levels of Blimp-1 and T-bet followed a similar pattern (Fig. 4f, p = 0.02, respectively), while Eomes expression did not differ significantly, neither for frequency of positive cells (p = 0.4) nor expression levels (p = 0.16). Taken together, liver- and peripheral blood-derived NK cells exhibited distinct expression patterns of transcriptions factors, and this was most apparent for liver-derived Hobit^pos^ CD56^bright^ NK cells.

## Discussion

Dissecting the factors that influence tissue-residency and shape the local immune microenvironment is crucial to improve our understanding of tissue-specific immunity. Several recent studies, mainly in mouse models, have demonstrated that liver-resident NK cells differ from NK cells circulating in the peripheral blood in expression of surface markers and transcription factors that regulate their trafficking behavior. In particular, the transcription factor Hobit has been shown to play a pivotal role in instructing tissue-residency of murine lymphocytes in conjunction with Blimp-1^14^. Hobit was furthermore shown to be expressed at high levels by peripheral blood NK cells^16, 17^ and iILC1s from human tonsils^18^. In this study we investigated Hobit expression by human NK cells in matched samples from peripheral blood and explant liver tissues. We demonstrate that CD56^bright^ NK cells in the liver expressed higher levels of Hobit compared to their blood counterparts, and that Hobit-expression was accompanied by an increased expression of tissue residency markers and changes in the expression of transcription factors.

In mice, the transcription factors Hobit and Blimp-1 acted synergistically to regulate the expression of a specific set of genes linked to tissue retention and egress^14^ of lymphocytes, thereby shaping tissue-specific immunity. Hobit/Blimp-1 double-knockout mice showed a down regulation of *Klf2*, *S1pr1*, *Tcf7* and *CCR7*, all genes previously liked to tissue egress or the formation of circulating immunity^19–23^. While Hobit was shown to be expressed by human NK cells circulating in the peripheral blood, it was almost absent in NK cells derived from human tonsils^16^, raising the question whether Hobit is also involved in regulating tissue-specific localization of human lymphocytes. A subsequent study showed that Hobit gene expression was high in iILC1 derived from human tonsils relative to other innate and adaptive lymphocyte subsets^18^. The differences observed in these two studies might be based on differences in the lymphocyte subsets studies, namely CD56+ NK cells^16^ versus CD56+ NKp44 + CD103+ iILC1s^18^. In line with the study by Vieira Braga *et al*.^16^, we observed Hobit expression by the majority of human NK cells circulating in the peripheral blood. Interestingly, Hobit was also expressed by NK cells derived from livers, in contrast to what had been described for NK cells derived from tonsils^16^. This demonstrates that expression of transcription factors by human NK cells differs between different tissues, as also suggested by recent studies of innate lymphoid cells in mice^24^.

While we did not observe differences in the frequency of Hobit^pos^ bulk NK cells or Hobit-expression levels among bulk and CD56^dim^ NK cells derived from peripheral blood or liver, CD56^bright^ NK cells obtained from liver tissues exhibited a significantly increased frequency of Hobit^pos^ cells and Hobit-expression levels compared to CD56^bright^ NK cells in the peripheral blood. These data suggest that CD56^bright^ NK cells, that have been shown to be enriched in tissues, might be required to reduce Hobit-expression in order to egress tissues and enter the peripheral circulation. However, the detection of Hobit^pos^ NK cells in the peripheral blood in humans in our study and the previous study by Vieira-Braga *et al*.^16^, while Hobit-expression is almost absent in circulating NK cells in mice, suggests clear differences in the function of Hobit between mice and humans. In humans, high Hobit-expression appears to be compatible with the circulation of CD56^dim^ NK cells in the peripheral blood, as also described for human CD45RA+ effector CD8 T cells, which express Hobit and circulate^16^, while Hobit-expression was reduce in CD56^bright^ NK cells circulating in the peripheral blood. In line with these differences in Hobit-expression in circulating lymphocytes between mice and humans, a number of Hobit-regulated genes are differentially expressed between mice and humans, including TCF1^25^, CD62L^26^ and granzyme B^27^. This indicates that different pathways may be required in mice and humans to facilitate tissue residency, and emphasizes the need to better understand the factors regulating tissue-entry, retention and egress of lymphocytes in humans. In this context it is important to highlight that the tissues used in our study were obtained from patients suffering from end-stage liver diseases of different etiologies, and might therefore not reflect the situation in healthy tissue. Despite the variability in the underlying diseases, we observed consistent differences in Hobit-expression between CD56^bright^ NK cells in livers and the peripheral blood, suggesting that Hobit-expression was involved in the determination of liver-residency of this NK cell subset.

The expression of several surface molecules involved in cell adhesion or chemotaxis have been associated with liver-resident NK cells in humans^9–12, 28^. Expression of CD49a, the alpha part of the α1β1 integrin complex which facilitates binding to collagen and laminin, is a critical marker for liver-residency in mice^8^, and was also shown to be almost exclusively expressed on human liver-derived NK cells and absent on peripheral blood NK cells^9^. CXCR6 has been described to be required to retain NK cells in the liver tissue^28^, and to be expressed by a subset of T-bet^lo^ Eomes^hi^ CD56^bright^ NK cells residing in the liver^10, 11^. Part of the Eomes^hi^ NK cells have been shown to persist up to 13 years in the liver in a transplantation setting^13^ and can be replenished by the Eomes^lo^ NK cells from the periphery. Very recently, the lack of CD49e was revealed as another possible marker to discriminate between tissue-resident and circulating NK cells^12^. We observed that Hobit^pos^ CD56^bright^ NK cells in livers contained an increased frequency of CD49a^pos^ and CXCR6^pos^ cells and that Hobit^pos^ CD56^bright^ NK cells in livers expressed higher levels of these markers associated with liver-residency on their surface compared to Hobit^neg^ CD56^bright^ NK cells and CD56^bright^ NK cells in the periphery. In addition, Hobit^pos^ CD56^bright^ NK cells also expressed higher levels of CD69, which has been described to be a hallmark marker of human tissue-resident memory T cells^29^ and NK cells^10^. CD49a expression showed the most pronounced difference between Hobit^pos^ and Hobit^neg^ CD56^bright^ NK cells in the liver, thereby being an important contributor to the tissue-resident phenotype. Differences in the expression of CD69 and CXCR6 were less dominant but still significant. These data suggest that Hobit^pos^ CD56^bright^ NK cells represent a tissue-resident population retained in livers by interactions with cell adhesion molecules and chemotaxis, in line with data obtained in Hobit/Blimp-1 knockout mice showing down-regulation of genes related to tissue egress^14^.

Expression of the transcription factor T-bet together with Eomes has been described for human liver-resident CD56^bright^ NK cells in several recent studies^9–11, 13^. In line with these studies, we observed that Hobit^pos^ CD56^bright^ NK cells in the liver expressed T-bet, which is, together with IL-15, involved in regulating the expression of Hobit^14^. Furthermore, while Eomes expression was lower among liver NK cells in general, Eomes expression was higher in Hobit^pos^ CD56^bright^ NK cells compared to Hobit^neg^ CD56^bright^ NK cells within the liver. In addition to expression levels also the frequency of Blimp-1^pos^ and T-bet^pos^ cells was increased among Hobit^pos^ CD56^bright^ NK cells. However, Hobit^pos^ NK cells in the liver did not include the T-bet^lo^ Eomes^hi^ subset of liver-resident NK cells recently described in two studies^10, 11, 13^, indicating that Hobit-positivity might define an independent subset of tissue-resident NK cells. Which might be predisposed to be recruited towards inflamed livers, due to the high expression of CD49a. One of the hallmarks of liver inflammation is fibrosis that is accompanied by an increase of collagen production, which acts as a ligand for CD49a^30^. CD49a^pos^ NK cells might therefore have a specific function in the context of liver inflammation, and it has been suggested that NK cells can prevent fibrosis by killing activated stellate cells^31^, one of the main drivers of liver fibrosis.

In conclusion, we show that the human liver harbors a subset of Hobit^pos^ CD56^bright^ NK cells expressing chemokine receptors and adhesion molecules associated with liver residency. Hobit^pos^ CD56^bright^ NK cells in livers expressed a distinct pattern of transcription factors differing from previously described intrahepatic NK cells. These data suggest that Hobit is involved in regulating tissue-residency of human intrahepatic CD56^brigt^ NK cells and that Hobit-expression can define a distinct subset of liver-resident NK cells in inflamed livers.

## Methods

### Study Design

The objective of this study was to investigate whether Hobit is expressed by human intrahepatic NK cells and whether it influences tissue-residency of NK cells. *Ex vivo* phenotyping using flow cytometry was performed on matched liver and blood samples from individuals who underwent liver transplantation. Randomization and blinding were not used in this study.

### Patient Cohort

Individuals undergoing liver transplantation at the Department of Hepatobiliary and Transplant Surgery in the University Medical Center Hamburg were included in this study. Reasons for transplantation varied and included alcoholic liver diseases (n = 2), hepatitis C virus infection (n = 2), hepatocellular carcinoma (n = 2) and Cholangiocarcinoma (n = 1). Informed consent was obtained from all patients. This study has been approved by the ethics committee of the Ärtzekammer Hamburg. All experiments were performed in accordance with the relevant guidelines and regulations. Matched blood samples were obtained, either during surgery or directly before. Samples of the explanted livers were collected during surgery, immediately transferred to the Heinrich Pette Institute and processed. Individuals undergoing repeated liver transplantations or individuals in whom no blood samples were obtained were excluded from the analysis.

### Sample processing

PBMCs were isolated from blood samples using density centrifugation with Ficoll/Percoll. Liver tissue samples were cut into small pieces using scalpels and subsequently mechanically dissociated using a gentleMACS Octo Dissociator (Miltenyi). No enzymes were used to assist with digestion of the tissue, due to their effects on the surface expression of NK cell markers. Liver tissue samples were subsequently serially filtered using decreasing filter sizes from 100 µm to 70 µm and 40 µm.

### Flow Cytometry

The following antibodies were used from Biolegend: anti-Blimp-1-PE, anti-CD16-BV785 (3G8), anti-T-bet-BV711 (4B10), anti-CD69-BV510 (FN50), anti-IgM-BV421 (RMM-1), anti-CD14-AlexaFluor700 (M5E2), anti-CD19-AlexaFluor700 (HIB19), anti-CD3-AlexaFluor700 (UCHT1), Zombie NIR Fixable Viability Kit, Zombie Aqua Fixable Viability Kit, anti-CXCR6-PerCP-Cy5.5 (K041E5), anti-CD56-BV605 (HCD56), anti-CD14-BV510 (M5E2), anti-CD19-BV510 (HIB19), LiveDead-Aqua, anti-CD45-APC-Cy7 (HI30), anti-CD3-BV510 (UCHT1). From BD Bioscience: anti-CD3-PE-CF594 (UCHT1), anti-CD45-BUV395 (HI30) and anti-CD56-BUV737 (NCAM16.2). Additional antibodies used were anti-CD49a-PE-Vio770 (Miltenyi), anti-Eomes-APC (R&D Systems) and anti-CD2-Qdot605 (S5.5, life technologies). The Hobit antibody was kindly provided by Klaas van Gisbergen^14^.

Stainings of cells (10^6^ PBMCs or 2 × 10^6^ liver-derived lymphocytes) were performed as follows: after washing in PBS with 2% (v/v) FBS and 0.5 M EDTA, cells were stained with an antibody mastermix prepared in the same buffer for 30 min at 4 °C in the dark. After two washing steps, cells were incubated in freshly prepared Fix/Perm solution (eBioscience) for 30 min at 4 °C in the dark. Following one washing steps, with freshly prepared Permwash (eBioscience), cells we stained with the Hobit antibody (1:10 dilution) for 30 min at 4 °C in the dark. Cells were subsequently washed two times and then stained with anti-IgM-BV421 for 30 min at 4 °C in the dark. After washing ones with both Permwash and PBS containing 2% (v/v) FBS and 0.5 M EDTA, cells were fixed in 4% (w/v) PFA. All samples were acquired on a BD LSRFortessa (BD Bioscience). The data was analyzed using FlowJo v10 Software (Treestar).

### Statistical Analysis

Statistical analyses were performed using Excel (Microsoft Corp.) and Prism 5 (GraphPad Software Inc.). Due to the small sample size, data were not assumed to be normally distributed and non-parametrical tests were performed. As matched peripheral blood and liver samples were obtained from the same individual, matched analyses were performed. Comparisons between two groups were performed using the Wilcoxon matched pair sign rank test to determine statistical differences. For comparisons between three or more groups, the Friedman test was used. If the p-value is not depicted as number it is abbreviated with *, where * represents p values below 0.05, ** below 0.01 and *** below 0.001.

## Data availability

The datasets generated during the current study are available from the corresponding author on reasonable request.

## Electronic supplementary material

Supplementary Information
