# Supplementary material for: Hobit expression by a subset of human liver-resident CD56bright Natural Killer cells
Source: Sci Rep. 2017 Jul 27;7:6676. doi: 10.1038/s41598-017-06011-7 (PMC5532267; doi:10.1038/s41598-017-06011-7)
Supplement: Supplementary file 1 — Supplementary Information [file 41598_2017_6011_MOESM1_ESM.pdf]

Hobit expression defines a subset of liver-resident CD56^{bright} Natural Killer cells in the human liver

Authors: Sebastian Lunemann[1†], Gloria Martrus[1†], Hanna Goebels[1], Tobias Kautz[1], Annika Langeneckert[1], Wilhelm Salzberger[1], Martina Koch[2], Madeleine Bunders[1], Björn Nashan[2], Klaas van Gisbergen[3], Marcus Altfeld[1*]

Table 1. Summary of clinical data for all patients used in the study.

Number	7
Gender (f/m; %)	1/6 (86%)
Age (years)	55 (25-63)
Weight (kg)	82 (64-132,5)
Size (cm)	176 (154-182)
AST (U/L)	38 (23-110)
ALT (U/L)	31 (15-85)
CMV (p/n, % positive)	4/3 (57%)

All clinical data is shown as median with minimum to maximum.
